# The Effectiveness of Triton Used With XP-3D Adaptive Files and Active Irrigation on Smear Layer Removal During Root Canal Preparation: A Qualitative Scanning Electron Microscopy (SEM) Study

**DOI:** 10.7759/cureus.73057

**Published:** 2024-11-05

**Authors:** Charissa Bandoo, Samiyah Suliman, Emma Trestrail, Shivaughn M Marchan

**Affiliations:** 1 School of Dentistry, The University of the West Indies, St. Augustine, TTO

**Keywords:** edta, root canal treatment, sem analysis, smear layer, triton

## Abstract

Background: Standard irrigating protocols during root canal treatment call for the use of sodium hypochlorite during chemo-mechanical preparation, followed by final irrigation with ethylenediaminetetraacetic acid (EDTA). This study determined the effectiveness of a new irrigant, Triton (Brasseler, USA), on smear layer removal during root canal treatment compared to that of 5.25% sodium hypochlorite and CanalPro EDTA 17% (Coltene).

Methodology: Twenty-one extracted anterior teeth were prepared for root canal treatment. The teeth were divided into three groups: five teeth were used as control with saline as the irrigant, eight teeth with sodium hypochlorite and 17% EDTA as irrigants, and eight teeth with Triton as the irrigant. Root canal preparation was done using the XP-3D shaper and finisher files (Brasseler, USA) for all teeth with respective irrigants. A single operator performed all root canal treatment procedures. The teeth were prepared for scanning electron microscopy (SEM), and analysis was performed by a second investigator. The smear layer removal was qualitatively assessed to determine the effectiveness of irrigants.

Results: Smear layer removal was most effective for teeth irrigated with 5.25% sodium hypochlorite, followed by 17% EDTA (Group B). Teeth irrigated with Triton (Group A) showed a similar appearance to the hypochlorite/EDTA group but with isolated instances of inter-tubular debris.

Conclusion: Sodium hypochlorite use followed by a final rinse with EDTA remains the gold standard for the removal of the smear layer created during canal instrumentation.

## Introduction

Mechanical preparation of the root canal system using endodontic files creates a smear layer that lines the walls of the canal [1,2]. This layer occludes the dentinal tubules, reducing the effectiveness of irrigants and intracanal medicaments used to disinfect the canal [3,4]. Additionally, the presence of the smear layer may affect the penetration of root canal sealers into the tubules [5].

Ethylenediamine tetraacetic acid (EDTA) has been shown to be effective at smear layer removal and has been used in conjunction with sodium hypochlorite (NaOCl) as an irrigation regimen [6]. EDTA demineralizes dentine and removes the inorganic components of the smear layer through its chelating effect [7]. Sodium hypochlorite is commonly used as the main irrigant throughout canal preparation because of its anti-microbial properties and the ability to dissolve necrotic pulpal tissue [8,9]. Following chemo-mechanical preparation of the root canal system with sodium hypochlorite, EDTA has been widely reported in the literature as a penultimate rinse for 1 minute [10,11]. This protocol has been widely accepted during root canal preparation.

Triton was developed to improve the efficiency of root canal treatment by formulating one irrigant that mimicked the physiochemical properties of both sodium hypochlorite and EDTA to dissolve the organic and inorganic components of the smear layer. It is a two-part solution that consists of two separate solutions. Part A contains a mixture of the following: 1,2,4-Butanetricarboxylic acid, 2-phosphono-, citric acid, sodium dodecylbenzene sulfonate, alcohols, C9-11, ethoxylated, liquids, polyethylene glycol 4-(tert-octylphenyl) ether, liquid, sodium lauryl sulfate, 2-Ethylhexyl sodium sulfate, sodium cumenesulfonate, sodium hydroxide. Part B is a mixture of sodium hypochlorite and sodium hydroxide. According to the manufacturer (Brasseler, USA), when both parts are mixed, the resulting solution has 4% sodium hypochlorite. This pilot study investigated and compared the effectiveness of the Triton to sodium hypochlorite and EDTA regimens on smear layer removal during root canal preparation.

## Materials and methods

An exemption of ethical approval (CREC-SA. 2254/7/2023) was granted for this study by the Ethics Committee of the University of the West Indies, St. Augustine. A total of 21 single-rooted, non-carious anterior teeth were used in this study. The teeth were randomly divided into three groups using different irrigants:

A. Eight teeth: Triton

B. Eight teeth: 5.25% sodium hypochlorite with a penultimate rinse for 1 minute with 17% EDTA

C. Five teeth: Saline (control group)

All teeth were accessed using conventional access cavity preparations. Working length for all teeth was determined by advancing a size 10 K-file (Kerr, Endodontics) through the apical foramen and then retracting the file by 1 mm. 

A size 15 K-file was used to prepare the canal to working length. This was followed by using an XP-3D shaper file (Brasseler USA) to determine the working length for each tooth, which was equivalent to an apical size of 30. The XP-3D finisher file (Brasseler, USA) was then used to determine the working length to complete the preparation. New XP-3D shaper and finisher files were used for each tooth at a speed of 800 rpm and a torque of 1 Ncm.

The irrigation protocol was as follows for the three groups:

A. Eight teeth were prepared using 10 ml of Triton irrigant throughout the canal preparation, followed by active irrigation for 1 minute with Triton using the EndoActivator (Dentsply, Sirona). The canal was finally irrigated with 2 ml saline.

B. Eight teeth were prepared as above using 8 ml 5.25% sodium hypochlorite irrigant throughout the canal preparation, followed by active irrigation with 2 ml 17% EDTA for 1 minute using the EndoActivator. The canal was finally irrigated with 2 ml saline.

C. Saline (10 ml) was used throughout canal preparation in five teeth (control group). At the end of canal preparation with the XP-3D Finisher, active irrigation was performed for 1 minute using the EndoActivator.

The teeth were decoronated at the level of the cemento-enamel junction. Grooves were placed longitudinally on the root surface, not involving the canal, using a water-cooled coarse diamond disc. The roots were split along the length using a chisel and mallet. The halves of each root were dehydrated in ascending concentrations of ethyl alcohol (30-100%). Immediately following alcohol dehydration, the specimens were placed in a desiccator for at least 24 hours. Each specimen half was mounted on an aluminum stub and qualitatively examined under a scanning electron microscope (Phillips SEM 515, Phillips, Eindhoven, Netherlands), operating at 30 kV and an operating distance of 12 mm. Photomicrographs were taken at X800 magnification and examined for overall smear layer coverage, dentine plugs, and inter-tubular debris.

Micrographs were obtained for the coronal, middle, and apical thirds of each of the irrigated canals. The researcher obtaining the SEM images (SM) was blinded to which irrigating solution was used on which teeth. The teeth provided to this researcher were only annotated with group labels A, B, and C, respectively. Only following the acquisition of the micrographs was the irrigating protocol revealed to this researcher. Representative micrographs are presented to demonstrate the qualitative descriptions presented in the results section.

## Results

The general observation of the micrographs at X800 magnification was that smear layer removal was most effective in Group B, followed by Group A and Group C, respectively. This pattern was also evident for all thirds of the roots examined. There was a preponderance of globular dentine in all the samples examined.

**Figure 1:**
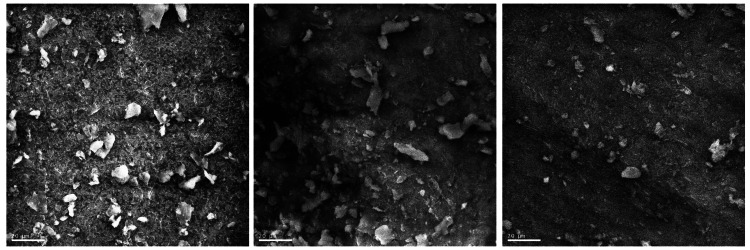
Micrographs of canals irrigated with saline Figures 1a-1c (from left to right) shows: Figure 1a. SEM image (X800) of the coronal third of a canal irrigated with saline. Figure 1b. SEM image (X800) of the middle third of a canal irrigated with saline. Figure 1c. SEM image (X800) of the apical third of a canal irrigated with saline.

The micrographs of Group C were teeth irrigated with saline. The smear layer was thick throughout the irrigated roots' coronal, middle, and apical thirds. There was no evidence of exposed dentine tubules throughout the entire length of the root canal (Figures 1a-1c).

**Figure 2:**
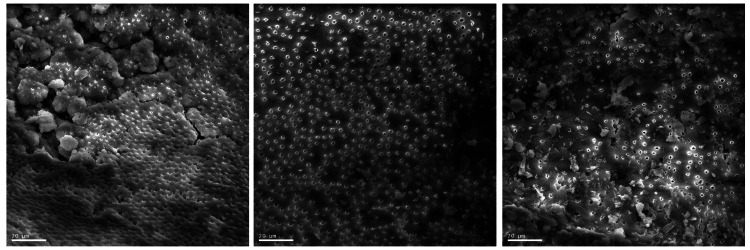
Micrographs of canals irrigated with Triton Figures 2a-2c (from left to right) shows: Figure 2a. SEM image (X800) of the coronal third of a canal irrigated with Triton. Figure 2b. SEM image (X800) of the middle third of a canal irrigated with Triton. Figure 2c. SEM image (X800) of the apical third of a canal irrigated with Triton.

There was significant smear layer removal along the length of the canals irrigated with Triton (Group A) compared with saline irrigation. Despite an overall reduction in the presence of a smear layer, there were isolated instances of inter-tubular debris noted in the Group A micrographs (Figures 2a-2c). 

This residual inter-tubular debris was less in the coronal and middle third of the canals compared with the apical thirds (Figure 2c), while the dentinal tubules appeared cleanest in the middle third of the root. 

**Figure 3:**
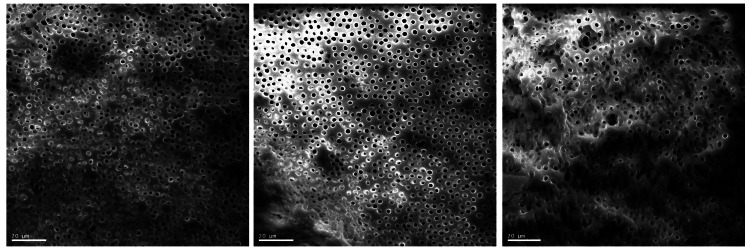
Micrographs of canals irrigated with sodium hypochlorite followed by EDTA Figures 3a-3c (from left to right) shows: Figure 3a. SEM image (X800) of the coronal third of a canal irrigated with sodium   hypochlorite/EDTA. Figure 3b. SEM image (X800) of the middle third of a canal irrigated with sodium hypochlorite/EDTA. Figure 3c. SEM image (X800) of the apical third of a canal irrigated with sodium hypochlorite/EDTA.

Teeth treated with 5.25% sodium hypochlorite and 17% EDTA (Group B) had a similar appearance to Group A with an overall absence of the smear layer compared along the entire length of the examined canals. There was a notable absence of inter-tubular debris in the canals treated with sodium hypochlorite and EDTA. The dentinal tubules appeared cleanest in the middle third of the root (Figures 3a-3c). 

The coronal third of Group B canals had a higher proportion of occluded dentinal tubules despite good overall smear layer removal (Figure 3b).

There was less inter-tubular debris in the apical third of canals irrigated with sodium hypochlorite and EDTA compared with canals irrigated with Triton (Figure 3c).

## Discussion

This study aimed to qualitatively assess the removal of the smear layer using an irrigating solution of Triton and compare it to the most widely used irrigation regimen of sodium hypochlorite and EDTA. Although the importance of smear layer removal on outcomes of root canal treatment is still debatable, there are known advantages of its removal [12-14]. Its removal allows for better penetration of irrigants, intra-canal medicaments, and obturation sealers into the dentinal tubules [5,15-17].

Saline lacks antibacterial and chelating properties and was therefore used as a control irrigant in this study [18]. It was evident that there was no effect on smear layer removal as the dentinal tubules were not evident along the entire length of the root canal system. This finding was expected given that recent research has shown saline to have the lowest efficacy on smear layer removal compared to other irrigating solutions [19].

The XP-3D shaper file system was used as opposed to traditional tapered nickel titanium files. This file system allows the maintenance of uniformity in canal preparation because of its ability to abrade the wide and narrow aspects of canals equally, taking into account the anatomical eccentricity of the canal space from the coronal to apical thirds [20,21]. This file system was used to approximate smear layer production across all the instrumented teeth given the natural variation in canal morphology [21].

In Groups A and B, the dentinal tubules in the middle third of the roots appeared cleanest. This was an unexpected finding given that dentine tubules are widest in the coronal third of the canal [22]. Given the larger volume of irrigant that should have been present in this widest coronal portion, it was anticipated that the coronal third of the canals would have had the least smear layer when compared to the middle and apical thirds. The authors concluded this finding might be due to a methodological error with the production of an additional smear layer during the decoronation of the teeth before SEM analysis. This methodological error is listed as the major limitation of this study. This extra smear layer production in the coronal third may have confounded the results but was mitigated by the examination of the entire length of the canals.

Another limitation is the sole use of single-rooted teeth with simpler endodontic anatomy compared to multi-rooted teeth. Given the limited research on Triton as an irrigating solution and its smear removal properties, further research is warranted on the use of Triton in multi-rooted canals with more complex anatomy and curved canals where teeth are decoronated prior to endodontic treatment.

The XP-3D Finisher file together with the EndoActivator was used for the active irrigation of all teeth with the selected irrigants. This reduced the challenges faced with passive irrigation only [23,24]. Active irrigation allowed sufficient irrigation of the apical anatomy in all the teeth, ensuring the interaction of irrigants with the canal walls in the apical third of the root [25,26]. Active irrigation is particularly effective in smear layer removal in the apical third of canals. [27]. Compared to saline, both irrigating protocols were effective in smear layer removal in the apical third of canals combined with active irrigation; however, the hypochlorite/EDTA irrigating protocol showed superior smear layer removal compared to Triton. This concurs with recent research, which demonstrated that Triton showed the lowest mean smear removal when compared with EDTA or chitosan nanoparticles as the final endodontic irrigant [28].

Sodium hypochlorite can dissolve organic components of the smear layer, while EDTA can dissolve inorganic components [29,30]. Given the effective concentration of sodium hypochlorite in Triton at 4% compared to the 5.25% of sodium hypochlorite, it can be theorized that the variance in the sodium hypochlorite concentrations played a role in the effectiveness of the removal of the organic components of the smear layer. Additionally, the combination of non-EDTA chelators and surfactants was less effective than 17% EDTA in the dissolution of the inorganic components of the smear layer.

Sheng et al. found Triton to be as effective as sodium hypochlorite and EDTA in smear layer removal [17]. However, in this study, smear layer removal was more effective in the middle and apical thirds of the canals irrigated with sodium hypochlorite and EDTA versus Triton. Given this comparative difference, the authors have postulated that EDTA is a superior chelating agent as opposed to the chelating agents present in the Triton solution. Although Triton has the advantage of a single irrigating solution, it is not as effective in smear layer removal as sodium hypochlorite followed by a penultimate rinse with EDTA [28]. Further clinical research into whether this impacts the outcome of root canal treatment would be beneficial.

Limitations of this study were the creation of an additional smear layer during the decoronation of teeth and the use of single-rooted teeth with simple endodontic anatomy compared to the more complex anatomy of multi-rooted teeth. Further work could include more teeth in the overall sample, followed by the use of imaging software to ascertain the percentage of patent tubules as a metric of smear layer removal. Quantitative statistical methods could then be used to ascertain differences in the effectiveness of the various irrigating solutions on smear layer removal, adding to the findings of this qualitative study.

## Conclusions

This qualitative study ascertained the effectiveness of a new, single-step irrigating solution, Triton, compared to saline (control) and sodium hypochlorite followed by EDTA. Single-rooted teeth were root-treated using rotary endodontic instruments. Each irrigating regimen (Triton or sodium hypochlorite) was used passively during canal preparation, and active irrigation was used at the end of canal preparation (Triton or EDTA). Teeth were decoronated, the roots split, desiccated, and examined using SEM for a qualitative evaluation of smear layer removal. Based on the findings of this qualitative assessment, the authors have concluded that sodium hypochlorite followed by EDTA irrigation was more effective at smear layer removal when compared with Triton in the middle and apical thirds of single canal teeth. However, compared to irrigation with saline, both irrigating regimens were more effective at smear layer removal. These findings must be cautiously interpreted given extra smear layer production during decoronation may have confounded the findings.
